# Characteristic of c-Kit^+^ progenitor cells in explanted human hearts

**DOI:** 10.1007/s00392-014-0705-3

**Published:** 2014-04-11

**Authors:** Sybilla Matuszczak, Justyna Czapla, Magdalena Jarosz-Biej, Ewa Wiśniewska, Tomasz Cichoń, Ryszard Smolarczyk, Magdalena Kobusińska, Karolina Gajda, Piotr Wilczek, Joanna Śliwka, Michał Zembala, Marian Zembala, Stanisław Szala

**Affiliations:** 1 Center for Translational Research and Molecular Biology of Cancer, Maria Skłodowska-Curie Memorial Cancer Center and Institute of Oncology, Gliwice Branch, Wybrzeże Armii Krajowej Street 15, 44-101 Gliwice, Poland; 2 Department of Cardiac Surgery and Transplantology, Silesian Center for Heart Diseases, M. Skłodowskiej-Curie Street 9, 41-800 Zabrze, Poland; 3 Foundation of Cardiac Surgery Development, Wolności Street 345a, 41-800 Zabrze, Poland

**Keywords:** Cardiac c-Kit^+^ cells, Progenitor cells, Mesenchymal stem cells, Explanted human hearts

## Abstract

According to literature data, self-renewing, multipotent, and clonogenic cardiac c-Kit^+^ progenitor cells occur within human myocardium. The aim of this study was to isolate and characterize c-Kit^+^ progenitor cells from explanted human hearts. Experimental material was obtained from 19 adult and 7 pediatric patients. Successful isolation and culture was achieved for 95 samples (84.1 %) derived from five different regions of the heart: right and left ventricles, atrium, intraventricular septum, and apex. The average percentage of c-Kit^+^ cells, as assessed by FACS, ranged between 0.7 and 0.9 %. In contrast to published data we do not observed statistically significant differences in the number of c-Kit^+^ cells between disease-specific groups, parts of the heart or sexes. Nevertheless, c-Kit^+^ cells were present in significant numbers (11–24 %) in samples derived from three explanted pediatric hearts. c-Kit^+^ cells were also positive for CD105 and a majority of them was positive for CD31 and CD34 (83.7 ± 8.6 and 75.7 ± 11.4 %, respectively). Immunohistochemical analysis of the heart tissue revealed that most cells possessing the c-Kit antigen were also positive for tryptase, a specific mast cell marker. However, flow cytometry analysis has shown cultured c-Kit^+^ cells to be negative for hematopoietic marker CD45 and mast cell marker CD33. Isolated c-Kit^+^ cells display mesenchymal stem cell features and are thought to differentiate into endothelial cells.

## Introduction

Dividing cardiomyocytes discovered in the human heart have challenged the paradigm of a myocardium built from terminally differentiated cells unable to proliferate [14]. Extensive research has led to identification of stem/progenitor cells in the heart. Several types of cells have been distinguished that show stem cell characteristics. Furthermore, many researches have performed studies with bone marrow-derived progenitor cells and circulating progenitor cells concerning its impact to postinfarction heart function. There have observed inconsistent effects on LV remodeling [9, 12, 20, 24].

One of stem/progenitor cells in the heart are cells with c-Kit receptor (CD117 or SCFR–stem cell factor receptor) on the surface. c-Kit^+^ cells proliferate both symmetrically and asymmetrically. Some of them demonstrate the expression of transcription factors typical of endothelial cells, vascular smooth muscle cells, or cardiomyocytes [3]. The c-Kit receptor is also found on the surface of hematopoietic stem cells as well as progenitor and mast cells, certain dendritic cells, and also on melanocytes [8]. Immunohistochemical analysis showed that more than 85 % of c-Kit^+^ cells observed in the cardiac tissue are mast cells [26].

According to Bearzi et al. c-Kit^+^ cells features seem typical of stem cells: they are self-renewing, clonogenic, and multipotent (able to differentiate into endothelial cells, smooth muscle cells and cardiomyocytes) [3]. Certain in vivo trial results indicate the capability of c-Kit^+^ cells to form a functional myocardium, and improve postmyocardial infarction function [3]. The results of this study laid foundation for Phase I randomized clinical trial Stem Cell Infusion in Patients with Ischemic cardiOmyopathy (SCIPIO), in which patients with heart failure resulting from ischaemic heart disease were treated with intracoronary infusion of autologous c-Kit^+^ cells proliferated under in vitro conditions. Preliminary results suggested improved contractile function of the left ventricle as well as reduced postinfarction scar size. There have been no deaths, cardiac cancer, or cardiac complications requiring hospitalization. This indicates that the transplantation of c-Kit^+^ cells is a safe method [5]. However, in recent years, some papers [16, 25] have contested the ability of c-Kit^+^ cells isolated from adult hearts to differentiate into cardiomyocytes; the ability to differentiate was displayed only by c-Kit^+^CD45^−^ cells isolated from the tissues of very young patients.

The transcriptome analysis and study of c-Kit^+^ cells’ ability to differentiate have indicated two classes of cells displaying the c-Kit marker: c-Kit^+^KDR^+^ cells (VPC, vascular progenitor cells) and c-Kit^+^KDR^−^ cells (MPC, myocyte progenitor cells) [2]. Among c-Kit^+^KDR^+^ cells are cells distinguished by the presence of CD31 marker [21]. The correctness of this distinction has also been confirmed by vascular endothelial stem cell research, in which a population of rare Lin^−^CD31^+^CD105^+^Sca1^+^CD117^+^ cells was described. The latter are capable of self-renewal and relevant to the process of angiogenesis [8]. Hematopoietic origin of these cells was excluded by research performed on both cardiac progenitor c-Kit cells and vascular stem cells [2, 8].

According to Gambini et al. c-Kit^+^ cells possess CD105 and CD29 markers which are typical of mesenchymal stem cells [11]. These cells can differentiate into typical MSC progeny lines, such as osteoblasts and adipocytes. However, their ability to differentiate is much lower when compared with bone marrow-derived MSC control cells [10, 11].

The aim of our research was to identify c-Kit^+^ cells in myocardial tissue and in cell cultures derived from explanted hearts obtained from recipients during transplantation.

## Materials and methods

### Ethical statements

This study was conducted according to the principles expressed in the declaration of Helsinki and was approved by the institutional review board.

### Patients and tissue samples

The material used in the study was explanted hearts removed during heart transplant surgery. Tissue fragments were harvested from right ventricle (RV), left ventricle (LV), intraventricular septum (IVS), atrium (A) and apex (APX). The material was collected from 19 adults and 7 children. The following general information was obtained about the patients: sex, age, and cardiovascular history. The collected material was used for immunohistochemical analysis and to establish in vitro cell cultures.

### Immunohistochemical analysis

The harvested material was fixed using 4 % paraformaldehyde and embedded in paraffin. Paraffin sections (5 μm) were obtained and incubated for 1 h in 4 % FBS (Foetal Bovine Serum, Gibco) to minimize nonspecific antibody binding. Next the sections were incubated with primary antibodies: anti-c-Kit (Dako), anti-Mast Cell Tryptase (Abcam) and, subsequently, with secondary antibodies conjugated with FITC (Dako) or Texas Red (Vector Laboratories). To reduce autofluorescence the tissues were incubated in Sudan Black B (Sigma) solution. Sections were then mounted using Mounting Medium with DAPI (to stain cell nuclei) (Vector Laboratories). The immunohistochemical sections were observed using a Zeiss LSM710 confocal microscope.

### Isolation and cell culture

Tissue material derived from RV, LV, IVS, A, and APX was cut into 1–3 mm^2^ pieces, washed with PBS^−^ (Mg^2+^, Ca^2+^-free phosphate buffered saline) to remove blood and fat tissue. Subsequently, the material was digested with collagenase IV (1 mg/ml, Sigma) for 10 min, and three times with 0.25 % trypsin (Sigma) for 5 min. The partially digested tissue fragments were incubated (37 °C, 95 % air and 5 % CO_2_) in IMDM medium (Sigma) supplemented with 20 % FBS, l-glutamine (Sigma), β-mercaptoethanol (Sigma), and antibiotics (penicillin and streptomycin, Sigma) and using culture plates coated with fibronectin (20 μg/ml). The medium was changed every 2 or 3 days for 3 weeks.

Human Umbilical Vein Endothelial Cells (HUVEC, ATCC) were grown (37 °C, 95 % air and 5 % CO_2_) in RPMI 1640 medium supplemented with 20 % FBS, heparin (100 μg/ml) and bFGF (50 ng/ml, BD Biosciences) and using culture flasks covered with 0.1 % gelatin. The medium was changed every 2 or 3 days.

### Phenotypic analysis

Upon reaching 70 % confluence, cells obtained from tissue fragment cultures were treated with 0.02 % EDTA solution and 0.05 % trypsin. A single cell suspension was obtained, and was next incubated (30 min at 4 °C) with suitable combinations of the following monoclonal antibodies against human antigens or isotype-matched control antibodies: c-Kit-APC, CD105-PE (eBioscience); CD31-FITC, CD34-FITC, CD45-APC-Cy7, CD33-PE, KDR-PE, Lin-FITC (BD Bioscience); finally analysed using a BD FACSCanto cytometer.

### Statistical analysis

Statistical significance of differences between groups was evaluated by ANOVA (analysis of variance). *P* < 0.05 was considered statistically significant.

## Results

**Fig. 1:**
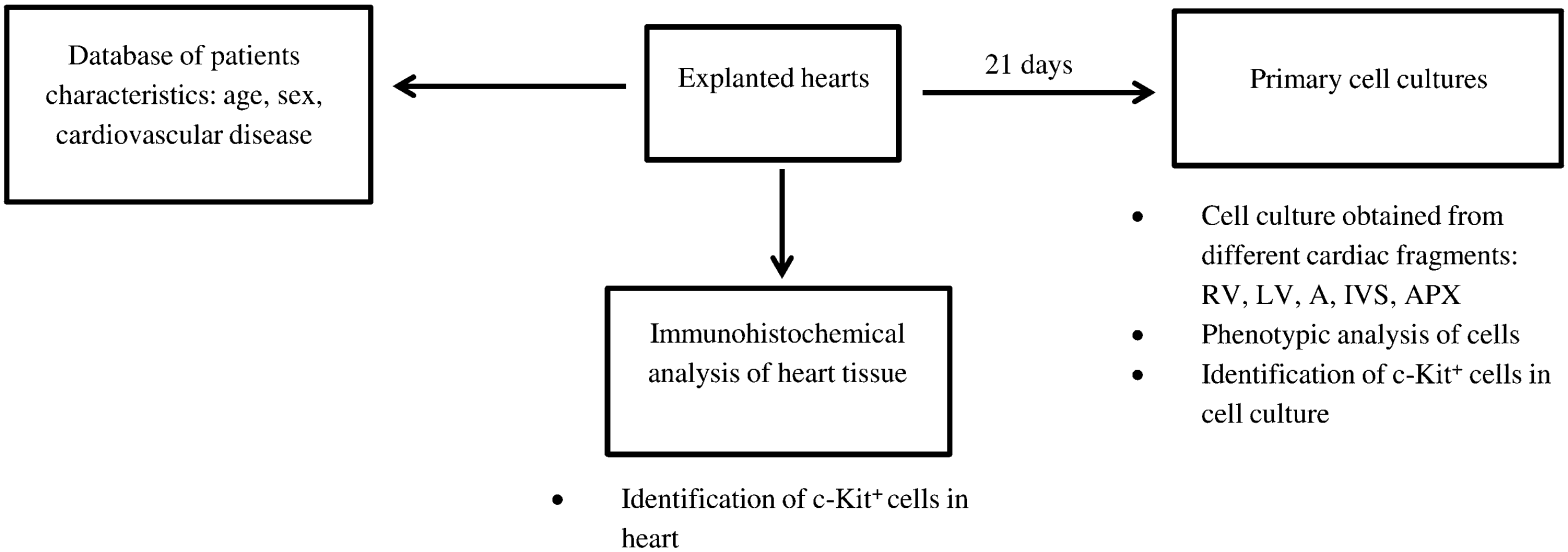
Experimental flow chart

The experimental flow chart for proceeding with the explanted material is shown in Fig. 1. Tissue fragments were used to establish primary cardiac cell culture. The remaining material was fixed for immunohistochemical analysis.

### Identification of c-Kit^+^ cells in the myocardial tissue

**Fig. 2:**
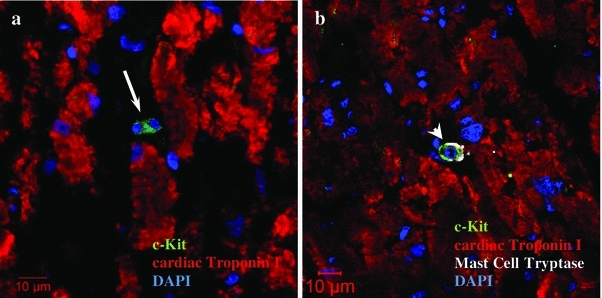
Identification of c-Kit^+^ cells in human heart. **a** The *arrow* indicates c-Kit^+^ (green fluorescence) progenitor cardiac cells, **b** the *arrowhead* indicates c-Kit^+^ (green fluorescence) tryptase^+^ (white fluorescence) mast cells. A few c-Kit^+^ tryptase^−^ cells were observed in the human cardiac tissue sections

A few c-Kit^+^tryptase^−^ cells were observed in the human cardiac tissue sections (Fig. 2a). Most of the c-Kit^+^ cells contained tryptase––an enzyme specific to mast cells (Fig. 2b). There were no differences in amount of c-Kit^+^ tryptase^−^ cells in the material derived from adults and children.

### Phenotypic analysis of cell cultures

**Fig. 3:**
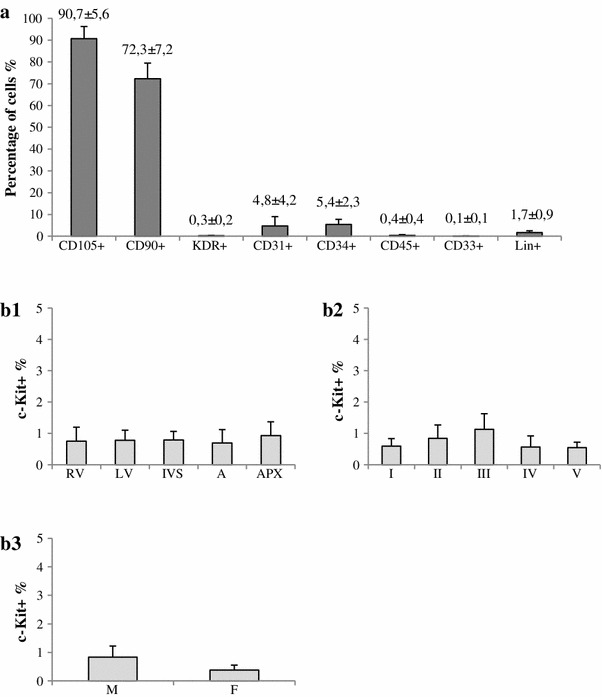
**a** Cardiac cell phenotype obtained in cell culture from adult patient material (*n* = 19). The majority of cells obtained in the culture had antigens typical of mesenchymal cells: CD105 and CD90. **b** The level of c-Kit^+^ cells in cultures from adult patient material (*n* = 19): **b1** the location of source tissue (*RV* right ventricle, *LV* left ventricle, *IVS* intraventricular septum, *A* atrium, *APX* apex), **b2** cardiovascular disease (*I* ischemic heart disease, *II* dilated cardiomyopathy, *III* hypertrophic cardiomyopathy, *IV* congenital heart defect, *V* others), **b3** patient’s sex (*M* male, *F* female). The level of c-Kit^+^ cells did not exceed 1 %

Cell culture was established for 95 (84.1 %) of 113 tissue fragments obtained from different cardiac regions (RV, LV, IVS, A, and APX). The material for cardiac cell culture was procured from 19 adult and 7 pediatric subjects (Tables 1, 2). Cardiac cells migrated from the cultured tissue fragments. After approximately 3 weeks, when at least 70 % confluency had been reached, an phenotypic analysis of cells was carried out (Fig. 3a). It showed that the majority of cells obtained in the culture had antigens typical for mesenchymal cells: CD105 and CD90 (90.7 ± 5.6 and 72.3 ± 7.2 %, respectively). The endothelial cells with CD31 and CD34 antigens accounted for a small percentage only (4.8 ± 4.2 and 5.4 ± 2.3 %, respectively). The culture did not contain any mast cells (CD33), hematopoietic cells (CD45), lineage markers (Lin), or progenitor endothelial cells (KDR). Percentage share of the above types of cells in cultures derived from various fragments of the heart, as well as from various patients remained similar.

**Table 1:** Characteristics of adult patients based on the age, sex, and type of cardiovascular disease

	Number of patients
**Age**	
18–34	1
35–49	6
50–65	12
**Sex**	
Male	15
Female	4
**Cardiovascular disease**	
Ischemic heart disease	7
Dilated cardiomyopathy	8
Hypertrophic cardiomyopathy	2
Congenital heart defect	1
Others	1

**Table 2:** Characteristics of pediatric patients based on age, sex, and type of cardiovascular disease and the percentage of c-Kit^+^ progenitor cells obtained in cell culture

Patient	Age	Sex	Cardiovascular disease	c-Kit^+^ cells (%)
1	11	Female	Dilated cardiomyopathy	13.7
2	12	Female	Noncompaction cardiomyopathy	1.9
3	3	Male	Dilated cardiomyopathy	24.1
4	14	Male	Hypertrophic cardiomyopathy	1.0
5	6	Female	Congenital heart defect	1.6
6	14	Male	Restrictive cardiomyopathy	Failed cell culture
7	14	Male	Restrictive cardiomyopathy	11.7

### Identification of c-Kit^+^ cells in in vitro culture

Cytometric analysis of cells obtained from in vitro cultures revealed that the level of c-Kit^+^ cells did not exceed 1 %. The level depended neither on tissue fragment origin (Fig. 3B1), past cardiovascular disorders (Fig. 3B2), nor the recipient’s gender (Fig. 3B3).

**Fig. 4:**
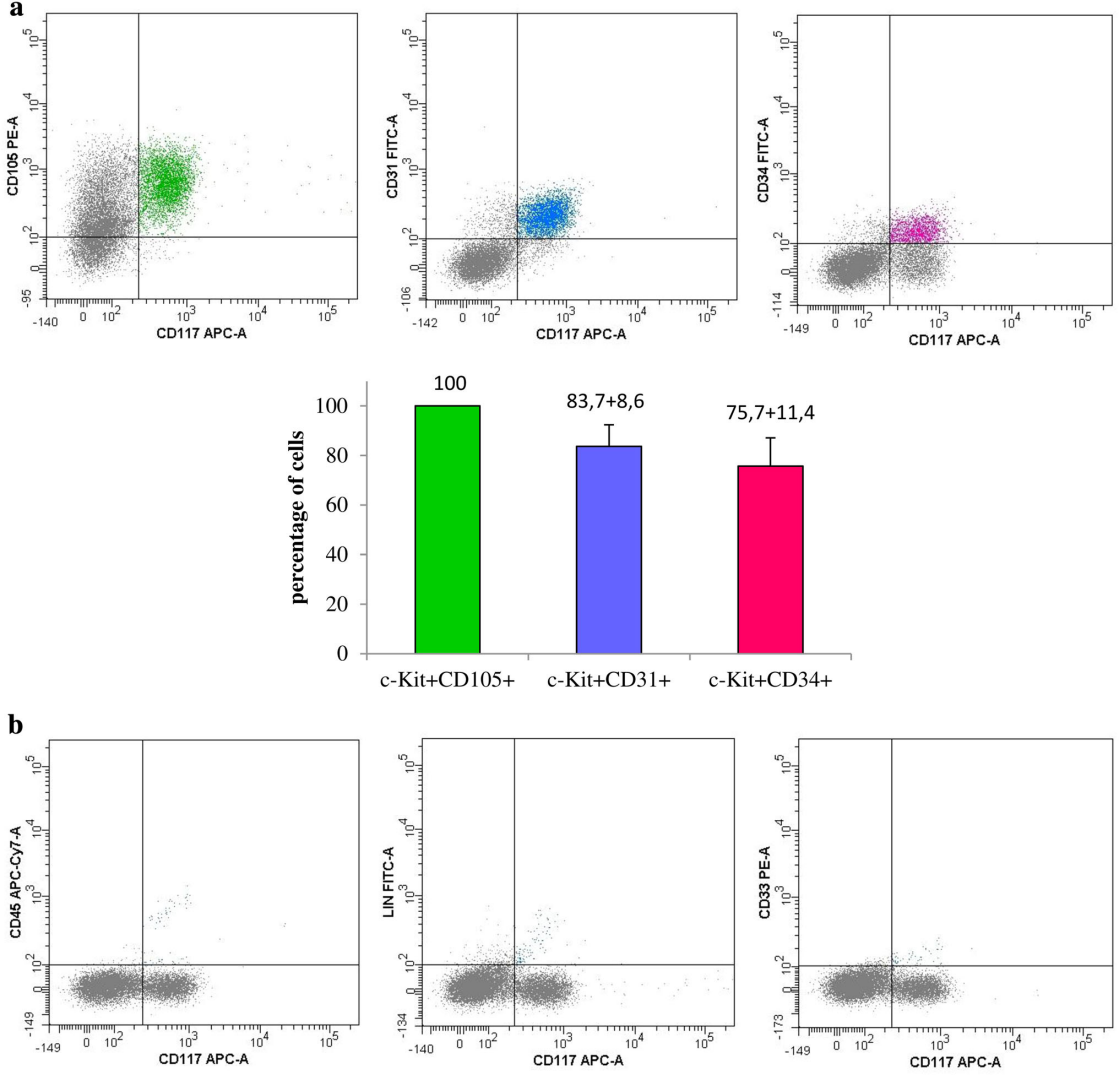
c-Kit^+^ cells in cell culture derived from pediatric patients’ (*n* = 3) material examined for: **a** CD105, CD31, and CD34 cells markers. CD105 mesenchymal cell marker was identified on all c-Kit^+^ cells; most of them contained endothelial cell markers. **b** CD45, Lin, and CD33 cells markers. c-Kit^+^ cells did not include any hematopoietic cell marker, lineage markers, or a mast cell marker

**Fig. 5:**
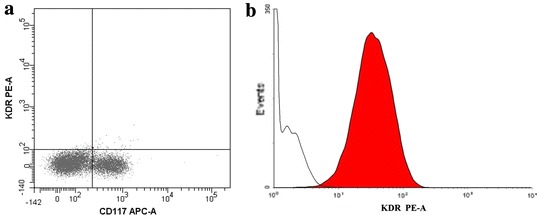
KDR progenitor endothelial cell marker: **a** c-Kit^+^ cells obtained in culture from pediatric patient (*n* = 3) material, **b** positive control (HUVEC cells). c-Kit^+^ cells did not include progenitor endothelial cell marker

An exception to this was the cultures obtained from part of the material derived from children. In cultures derived from three pediatric subjects, c-Kit^+^ percentage ranged from 11 to 24 % (Table 2). These cells had neither CD45 hematopoietic cell marker, nor lineage markers (Lin) or CD33 mast cell marker (Fig. 4b). c-Kit^+^ cells obtained from in vitro culture did not possess KDR surface marker of progenitor endothelial cells (Fig. 5a). However, CD105 mesenchymal cell marker was identified on all c-Kit^+^ cells (Fig. 4a). Furthermore, majority of cells showed also CD31 and CD34 endothelial cell markers (83.7 ± 8.6 and 75.7 ± 11.4 %, respectively).

## Discussion

Since c-Kit^+^Lin^−^ cells, considered to be resident cardiac stem cells, were discovered in human heart muscle [3] numerous research papers have focused on identification, in vitro characterization and potential applications of stem cells in the regeneration of damaged myocardium [6, 13, 15, 19].

Our phenotypic analysis of cell cultures grown from cardiac fragments showed that the main population consists of cells with CD105 and CD90 mesenchymal antigens. The cardiac cell culture obtained appeared immunophenotypically similar to that described by Davis et al. [7]. The culture included a small population of c-Kit^+^ cells (<1 %). Data found in the literature indicate a relationship between the number of c-Kit^+^ cells and their location in the heart [13, 19]. Both immunohistochemical analyses [17] and in vitro cultures derived from the right atrium [13] suggest that it is a source of greatest numbers of c-Kit^+^ cells. However, our data do not support this observation. Similar levels of c-Kit^+^ cells, not exceeding 1 % (0.7–0.9 %), were observed in cell cultures derived from different cardiac fragments of adult individuals. Itzhaki-Alfia et al. found a greater number of c-Kit^+^ cells in cultures grown from women’s hearts [13]. Our data do not support this observation neither. Some studies have indicated an increased number of c-Kit^+^ cells in patients with acute cardiac failure [10, 15, 23]. Nevertheless, our research did not find past cardiovascular diseases to affect the number of c-Kit^+^ cells in cardiac cell culture. Our observations are consistent with the data provided by Aghila Rani et al. [1]. However, we had at our disposal no control tissue from healthy subjects, the characteristics of which would allow us in determining increased numbers of c-Kit^+^ cells in connection with advanced stage of cardiac disease.

In tissue cultures derived from pediatric material (children aged 3–14), c-Kit^+^ cells ranged between 1 and 1.9 % of total number of cells with the exception of three children (11.7–24.1 %). These results are consistent with the observations of Mishra et al. suggesting that child’s cardiac tissue contains more c-Kit^+^ cells [17]. The increased number of c-Kit^+^ cells in these three pediatric cases may be caused by the inflammation processes occurring in heart’s tissue. Some literature data suggest the existence of two classes of c-Kit^+^ cells: c-Kit^+^KDR^+^ and c-Kit^+^KDR^−^, lacking CD105, CD90, CD45, CD133, and CD34 markers [2, 6]. The c-Kit^+^ cells isolated by us did not show the presence of KDR marker. All c-Kit^+^ cells had a CD105 marker and most of them possessed CD31 and CD34 markers as well. The presence of CD105 marker on c-Kit^+^ cells may suggest their mesenchymal origin. Gambini et al. showed that mesenchymal cells markers (CD105, CD44, CD29) are found on c-Kit^+^ cells which differentiate to adipocytes and osteoblasts [11]. It seems that c-Kit^+^ cells may have phenotypic and functional features of cardiac-specific mesenchymal cells [11]. Fang et al. identified a vascular endothelial stem cells (VESC) cell population with Lin^−^CD31^+^CD105^+^Sca1^+^CD117^+^ phenotype [8]. It is possible that c-Kit^+^CD105^+^CD31^+^Lin^−^ cell population identified in the child’s heart may participate in angiogenesis. D’Amario et al. obtained a population of c-Kit^+^ cells from biopsy specimens derived from failed hearts, after ca. 40 days of in vitro culture [6]. In small animals, within ca. 7 days from myocardial infarction, inflammation recedes resulting in diminished number of factors involved in the chemotaxis of the cells to the site of injury, factors affecting differentiation and cell’s survival [22]. Therefore, long culture time to obtain adequate numbers of c-Kit^+^ cells may adversely affect the therapeutic effectiveness of transplanted cells.

Low number of c-Kit^+^ cells obtainable in culture is consistent with low numbers of these cells in the cardiac tissue (1 per 10^4^ myocytes) [4]. Pouly et al. showed that all c-Kit^+^ cells identified in the cardiac tissue possess a CD45 marker indicating their hematopoietic origin. Additional staining for tryptase (a specific mast cell marker) confirmed the presence of these cells [19]. Increased number of mast cells in the damaged heart corresponds to greater number of c-Kit^+^ cells [18]. Lack of additional staining for mast cells’ presence can overrate the number of cells referred to as progenitor c-Kit^+^ cells. Other papers have reported that not all c-Kit^+^ cells are mast cells [15, 26]. This finding is in line with our observations that only single progenitor c-Kit^+^ cells are present in cardiac tissue.

Despite preliminary positive results of SCIPIO clinical trial, there have been critical reports negatively assessing the ability of c-Kit^+^ cells to rebuild the postinfarction myocardium. The ability of c-Kit^+^ cells to differentiate into cardiomyocytes, both in vitro and after transplantation to the damaged myocardium, has been challenged [25]. We did not provide any evidences standing for or against acquisition by c-Kit^+^ cell “cardiac like” phenotype. In our study, in descriptive manner, we characterized c-Kit^+^ cells as cells displaying mesenchymal stem cells features with mostly endothelial specification. More convincing study need to be done to confirm their ability to differentiate into cardiomyocytes. At present, it is believed that for the therapeutic effect observed after implanting cells to the postinfarction myocardium, responsible are factors secreted by these cells (the so-called paracrine effect). According to Tang et al. [22] direct regeneration of postinfarction heart by exogenous c-Kit^+^ cells is poor. However, the observed improvement of the structure and function of the left ventricle is rather mediated by a paracrine effect of cytokines produced by the implanted cells. These cytokines stimulate endogenous c-Kit^+^ cells. It is supposed that this effect can be maintained even after elimination all of implanted c-Kit^+^ cells.

## Limitations

We are aware of the obvious limitation of our study. The material was collected from patients of different age, gender and cardiovascular disease. This variables might have impact on the obtained cell culture. The phenotype of cultured cells is also strongly influenced by in vitro conditions. This plasticity may cause that cells will acquire or lose characteristic antigens. Cells description based on the presence of surface markers is a phenomenal study. We can only observe cells in exact time and conditions. Thus, in vitro culture will not reflect phenotype of cells in vivo.
